# Malignant germ cell tumor of fallopian tube with rhabdomyosarcoma: a case report and literature review

**DOI:** 10.1186/s13000-023-01388-8

**Published:** 2023-09-02

**Authors:** Liubiqi Zhao, Yan Li, Fang Wang, Xi Zheng, Li He, Lushuang Zhang

**Affiliations:** 1 Department of Obstetrics and Gynecology, Chengdu Women’s and Children’s Central Hospital, School of Medicine, University of Electronic Science and Technology of China, Chengdu, China; 2 Department of Pathology, Chengdu Women’s and Children’s Central Hospital, School of Medicine, University of Electronic Science and Technology of China, Chengdu, China

**Keywords:** Germ cell tumor, Fallopian tube malignancy, Rhabdomyosarcoma

## Abstract

**Background:**

Mixed germ cell tumors originating from the fallopian tubes are rarely reported, and high-grade rhabdomyosarcoma with differentiated components is even less common. The non-specific clinical manifestations of this tumor are prone to misdiagnosis, and there is still controversy over the treatment plan for this rare differentiated type, and there are limited reports on the prognosis of related diseases.

**Case presentation:**

Here, we report a 34-year-old woman who presented to our hospital with abdominal pain for two weeks and aggravated for two days. After completing relevant examinations, she underwent transabdominal resection of large tubal masses on the left side of the tube + pelvic lymph node dissection + abdominal paraaortic lymph node dissection + right ovarian cyst excision + greater omentectomy + multipoint peritoneal biopsy. hematoxylin–eosin (H&E) and immunohistochemical (IHC) staining were performed on the surgically resected specimens to further determine the type and nature of the tumor, and 3 cycles of Bleomycin + Etoposide + Cisplatonum (BEP) chemotherapy and 1 cycle of EP(Etoposide + Cisplatonum) chemotherapy were given after surgery.

**Conclusion:**

Up to now, regular follow-up of the patient's tumor markers and imaging showed no abnormalities, the general condition is good, and the tumor free survival time has reached 24 months.

## Background

Despite the great similarity in diagnosis and treatment between fallopian tube malignancies and ovarian tumors, the researchers show that there are still differences in biological origin and prognosis. The incidence of mixed germ cell tumor of fallopian tube combined with specific differentiation components is very low, and the treatment of this disease should be referred to the treatment of ovarian malignant germ cell tumor. However, whether its specific differentiation type requires further treatment, and the treatment mode, including the prognosis, is still unclear.

## Case report

A 34-year-old Chinese female, due to lower abdominal pain for 2 + weeks, aggravation for 2 + days, no other abnormal manifestations, gynecological B-ultrasound examination indicated “pelvic cavity mixed giant mass, pelvic cavity effusion”, urine HCG weak positive. During physical examination, a mass about 20 + cm in diameter could be touched in the middle and lower abdomen, the quality was hard, the motion was poor, there was deep tenderness, no rebound pain, and the muscle tension was positive.

**Fig. 1:**
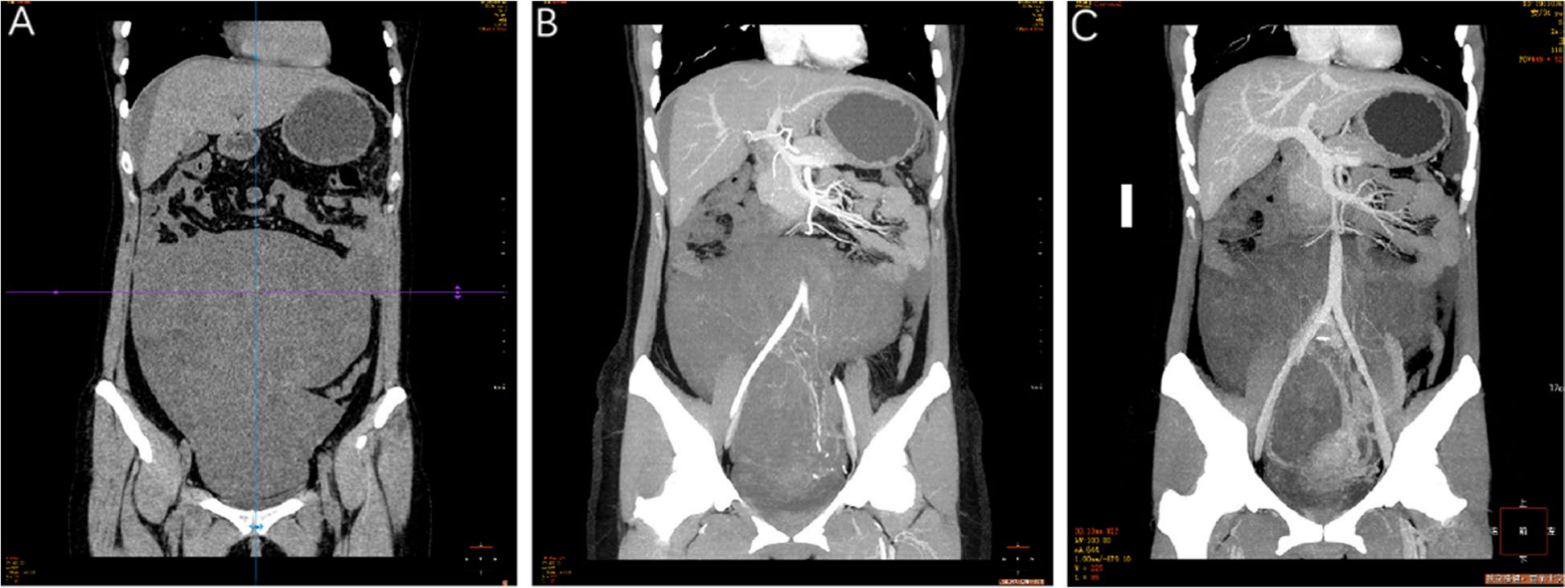
The computed tomography scan of pelvic cavity. **A** (CT plain scan) Huge mass occupying the pelvic cavity, the size of about 8.1*22.3*25.8 cm, its internal density is low, slightly mixed. **B** (CT enhancement (venous phase)) The enhancement was not obvious, and the huge pelvic occupying vein joined the left ovarian vein. **C** (CT enhanced (arterial phase)) Huge pelvic mass supplied by the left ovarian artery

Tumor markers: Carcinoembryonic antigen (CEA): > 100.00 ng/ml, alpha-fetoprotein (AFP): > 1000.0 ng/ml, carbohydrate antigen 125(CA125): > 600.0U/ml, carbohydrate antigen 199(CA199):554.63U/ml. Lactate dehydrogenase(LDH):718.7U/L, α-hydroxybutyrate dehydrogenase:625.7U/L. Human chorionic gonadotropin (HCG):33.2mIU/ml. Transvaginal color Doppler ultrasonography showed that the metrorectal depression was found to be 8.8cmx4.3 cm free and echoless. A heterogeneous weak echo mass of 17.5cmx8.2cmx25.5 cm was found in the middle and lower abdomen and scattered cystic echoes were found inside. Abdominal CT plain scan + enhancement (Fig. 1): huge tumor in the middle and lower abdominal cavity and pelvis. Considering the possibility of left adnexal origin, epithelial-derived mucocarcinoma, other ovarian tumors to be drained, greater omentum and peritoneum involvement, and abdominal fluid accumulation; suspiciously enhanced nodules at lower left, not excepting peritoneal etastases or false peritoneal mucous.

Considering the patient’s tumor biomarkers and CT results, it is highly likely that a large mass in the pelvic and abdominal cavity is a malignant tumor. After improving the preoperative preparation, transabdominal exploration was performed. Intraoperative exploration revealed that there was a huge mass in the pelvic cavity, about 24*22*9 cm in size, originating from the left fimbrium of the fallopian tube. The mass was cystic and carrion, with uneven surface and local laceration. Dark brown ascites can be seen, the amount is moderate; The greater omentum was partially thickened, and miliary nodules were found on the surface of pelvic peritoneum, especially in the fold of vesical peritoneum. The left fallopian tube and the huge mass were removed completely and sent for frozen section, weighing about 2.94 kg. Frozen section showed: (huge mass in pelvis and abdominal cavity) tumor with complex composition, mainly sarcomatoid stromal composition, interspersed with moderate atypical epithelial components, the type is difficult to determine, and the possibility of malignancy is high. During the operation, it was considered that the patient was fallopian tube malignant tumor with complex components and high malignancy degree, and extensive abdominal metastasis was not excluded. Pelvic lymph node dissection + para-aortic lymph node dissection + right ovarian cyst exfoliation + greater omentectomy + multipoint peritoneal biopsy was performed, and follow-up treatment plan was determined after H&E and IHC staining.

**Fig. 2:**
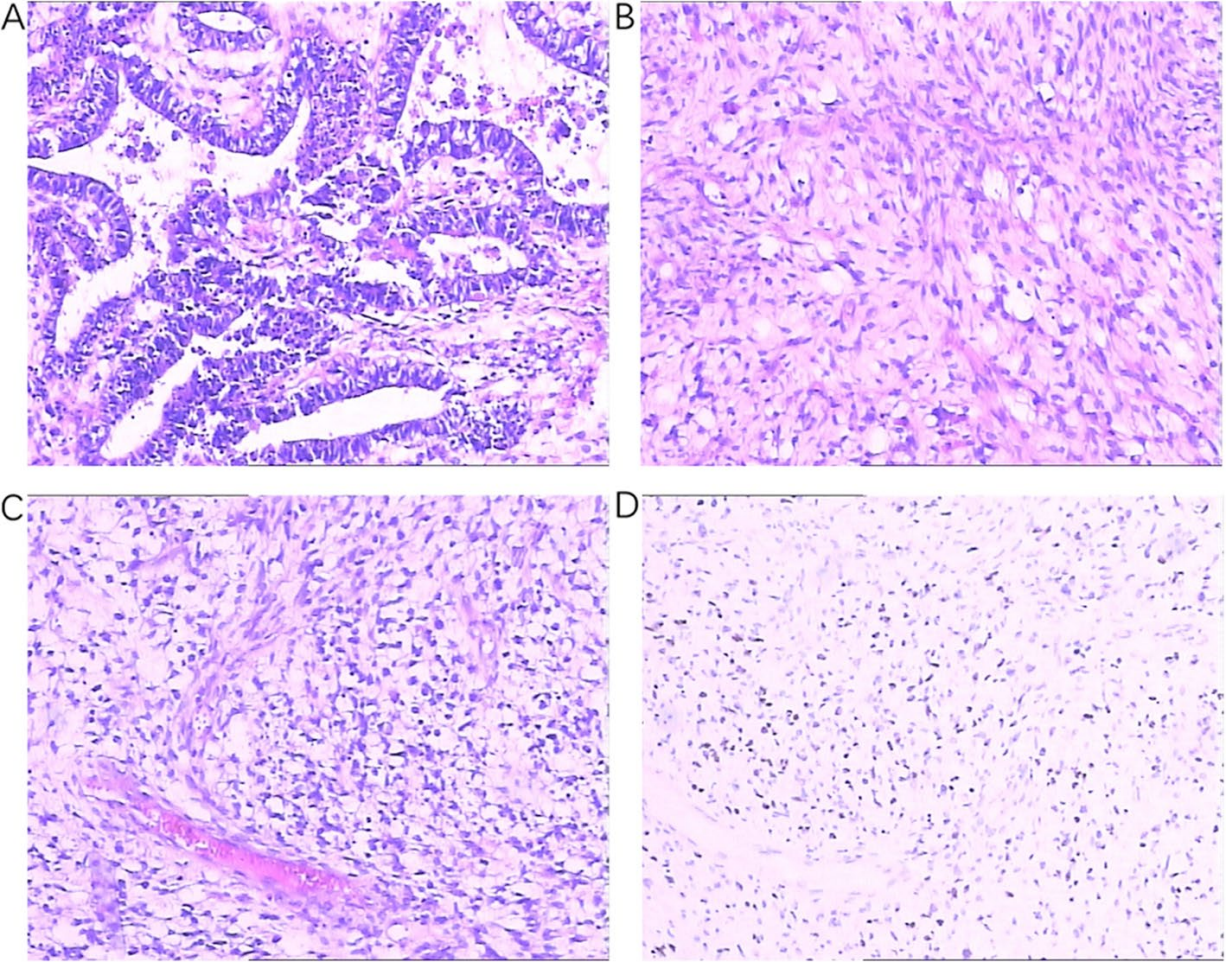
Images of excised tumor tissue. **A** Microscopic image of the differentiated tissue type of a vitelline sac tumor (H&E × 100). **B** Microscopic image of the sarcomatous tissue type of the tumor (H&E × 100). **C** Microscopic image of the sarcomatous tissue type of the tumor (H&E × 100). **D** Immunohistochemical image of MyoD1 in Fig. 2C. Sarcomatoid area ( +)

Postoperative pathology (Fig. 2): (huge pelvic mass) mixed germ cell tumor, the main component was yolk sac tumor, focal immature teratoma; Multiple areas of high-grade sarcoma (rhabdomyosarcoma). A small amount of tumor was found in the peritoneum. (Immunohistochemistry: 7#APF( +); 11#SALL4(-), Otc-4 (epithelium +), pCK(epithelium +), Vimentin (Sarcoma +), SMA (sarcomatoid region +), Desmin (Sarcomatoid region +), Caldesmon (Sarcomatoid region -), MyoD1(sarcomatoid region +), Ki-67 (+ , About 50%); 20 # Pax—8 (-), PLAP ( +), APF ( +), CD30 (-), EMA ( +), pCK ( +), SMA ( +), Otc—4 + (part), Ki—67 (+ , about 50%).

Postoperative diagnosis was clear: left fallopian tube mixed germ cell tumor (yolk cyst tumor + immature teratoma) with high-grade rhabdomyosarcoma stage II B. Considering that the patient had already given birth, and the tumor composition was complex and the malignancy was high, the multidisciplinary team of gynecologic oncology in our hospital decided to follow the treatment plan of malignant germ cell tumor of the ovary and plan to perform a second operation (comprehensive staging operation: total hysterectomy + bilateral oophorectomy + right salpingotomy + suspicious lesion resection). The examination after the second operation revealed that tumor (teratoma component) was found in a small amount of fibrous connective tissue (bladder surface peritoneal tissue). There was no tumor involvement in uterus and cervix, right fallopian tube and bilateral ovaries. Intraperitoneal thermoperfusion was performed with the following schemes: normal saline was injected 1–2 days after surgery, cisplatin was injected on the third day after surgery, and bleomycin + etoposide + cisplatonum (BEP) was started on the seventh day after surgery. A total of three circles of BEP were given. And one course of EP regimen (the last course of bleomycin caused interstitial pneumonia, and bleomycin was discontinued).

**Fig. 3:**
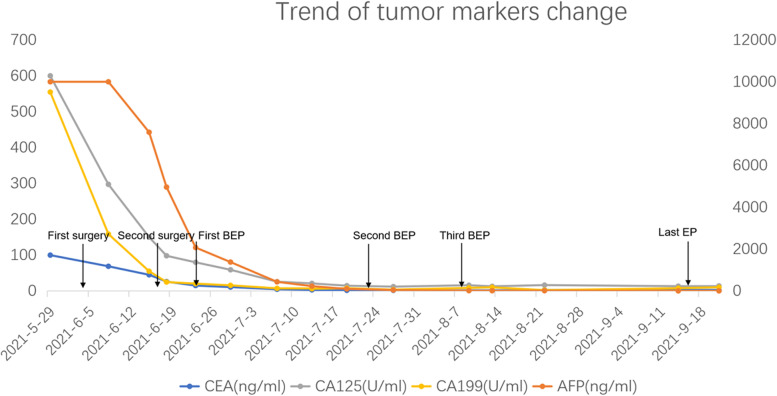
Trend of tumor markers change during treatment

The tumor markers of the patient decreased significantly after surgery compared with those before surgery, and further decreased with the smooth progress of the chemotherapy cycle. After the third BEP chemotherapy, the tumor markers decreased to total negative (Fig. 3). The patient’s quality of life was still satisfactory after follow-up, and no tumor recurrence was observed.

## Discussion

Germ cell tumors reflect the diverse potential of the original cells to differentiate into all somatic cells (endoderm, mesoderm, ectoderm) and extra-embryonic tissues. It is because of the differentiation potential of germ cell tumors as described above that we can also understand the multiple differentiation components shown in the pathology of this patient. Usually, the symptoms of MOGCT are non-specific and may overlap with those of epithelial ovarian cancer. MOGCT can lead to elevated tumor markers in peripheral blood, including AFP, β-hCG and LDH [1], although there are many factors affecting these tumor markers, However, it can still have a great auxiliary role in our diagnostic screening, treatment effect evaluation and early detection of recurrent cancer.

As with epithelial ovarian cancer, the staging of MOGCT is determined surgically, which is critical for assessing prognosis, follow-up, and treatment [2]. Whether to perform routine lymph node dissection is still controversial, depending on the tumor subtype and preoperative and intraoperative findings [3–5]. Although routine secondary surgery is not recommended, patients who have not completely removed the tumor in the initial surgery and have teratogenic components in their primary tumor may benefit from secondary cell reduction surgery [6]. First-line chemotherapy for MOGCT includes BEP, which, while highly effective, may cause significant short—and long-term side effects. At present, genomic studies on MOGCT are insufficient, and such genomic analysis is limited by the huge heterogeneity of tumors within each group. Existing studies have shown that the most common mutated genes are KIT and KRAS, and our cases were not examined for genetic testing due to personal factors.

The pathology of this patient has a high-grade rhabdomyosarcoma component, which is the most common soft tissue sarcoma in children and represents an advanced tumor of skeletal myoblast-like cells, a type of advanced malignancy in which cancer cells have a tendency to myogenic differentiation. Treatment should begin with the eradication of the gross primary tumor, as well as chemoradiotherapy, which recommends vincristine, actinomycin D and cyclophosphamide. In the course of our treatment, experts recommend two additional courses of chemotherapy, depending on the composition of the patient's rhabdomyosarcoma. We have reviewed relevant reports, and it is indeed mentioned in the literature that the ovarian teratoma contains differentiation components in other parts and needs further treatment. We have found relevant literature reporting that prophylactic total thyroidectomy, radioactive iodine ablation (RAI) and levothyroxine suppression should be given to ovarian teratoma containing thyroid tissue [7]. And many scholars believe that the treatment of ovarian teratoma with papillary thyroid carcinoma should be treated as thyroid cancer rather than ovarian cancer. In combination with the above, we offer patients the option to try further chemotherapy targeting the components of rhabdomyosarcoma. The patient refused this regimen due to adverse reactions after BEP chemotherapy and personal factors. This also provides us with diagnostic and treatment ideas for the treatment of this rare differentiation type, whether it is necessary for us to organize into branches for further treatment of its rare differentiation.

## Conclusion

In this case, the patient accidentally found a huge pelvic tumor during the examination due to the symptoms of abdominal pain. Prior to this, the patient had not undergone physical examination for a long time and had no symptoms. In the course of diagnosis and treatment, the huge size of the tumor, the significant increase of tumor markers, and the hints of imaging examinations alerted us of the possibility of malignant tumor before surgery. As mentioned above, the low elevation of human gonadotropin and the significant increase of AFP and LDH suggest the possibility of malignant germ cell tumor. In the course of treatment, we performed comprehensive staged surgery according to the MOGCTs treatment, and gave abdominal heat perfusion therapy and BEP chemotherapy after surgery. However, the side effects of chemotherapy drugs during the treatment also sounded the alarm for our future treatment. In our future research, we can avoid serious side effects through more and more means. The ideal decline of tumor markers in patients after surgery, as well as the present good quality of life, provides us with a favorable reference for the treatment of fallopian tube malignant germ cell tumors, and also allows us to have a basic grasp of the prognosis of related diseases.

## Data Availability

Not applicable.

## Abbreviations

H&E: Hematoxylin–Eosin
BEP: Bleomycin + Etoposide + Cisplatonum
IHC: Immunohistochemical
CEA: Arcinoembryonic ntigen
AFP: Alpha-Fetoprotein
CA125: Carbohydrate antigen 125
CA199: Carbohydrate antigen 199
HCG: Human chorionic gonadotropin
LDH: Lactate dehydrogenase
MOGCT: Malignant ovarian germ cell tumor

## References

[1] Parkinson CA, Hatcher HM, Ajithkumar TV (2011). Management of malignant ovarian germ cell tumors. Obstet Gynecol Surv.

[2] Palenzuela G, Martin E, Meunier A, Beuzeboc P, Laurence V, Orbach D (2008). Comprehensive staging allows for excellent outcome in patients with localized malignant germ cell tumor of the ovary. Ann Surg.

[3] Liu Q, Ding X, Yang J, Cao D, Shen K, Lang J (2013). The significance of comprehensive staging surgery in malignant ovarian germ cell tumors. Gynecol Oncol.

[4] Brown J, Sood AK, Deavers MT, Milojevic L, Gershenson DM (2009). Patterns of metastasis in sex cord-stromal tumors of the ovary: can routine staging lymphadenectomy be omitted?. Gynecol Oncol.

[5] Mahdi H, Swensen RE, Hanna R, Kumar S, Ali-Fehmi R, Semaan A (2011). Prognostic impact of lymphadenectomy in clinically early stage malignant germ cell tumour of the ovary. Br J Cancer.

[6] Gershenson DM (2007). Management of ovarian germ cell tumors. J Clin Oncol.

[7] Rockson O, Kora C, Ramdani A, Basma A, Bouhout T, Serji B (2020). Struma ovarii: two case reports of a rare teratoma of the ovary. J Surg Case Rep.

